# Diagnostic value and role of serum miR-15a-5p in patients with schizophrenia

**DOI:** 10.1186/s12991-023-00489-4

**Published:** 2024-01-05

**Authors:** Zhen Xu, Ruidong Yang, Guanwen Chen, Mingjun Jiang

**Affiliations:** 1 Department of Anesthesiology, The Eighth Medical Center of PLA General Hospital, Beijing, 100091 China; 2 Department of Adult Cardiovascular Surgery, The Sixth Medical Center of PLA General Hospital, Beijing, 100048 China; 3 Guangdong Nantian Institute of Forensic Science, No. 5003 Binhe Road, Futian District, Shenzhen, 518033 Guangdong China; 4 Shenzhen Polytechnic University, No. 7098 Liuxian Avenue, Nanshan District, Shenzhen, 518055 Guangdong China

**Keywords:** Schizophrenia, MiR-15a-5p, PANSS, MK-801

## Abstract

**Background:**

More and more studies have confirmed that the heredity plays an important role in mental disorders, especially microRNA. The objective of this research was to explore the level of miR-15a-5p in patients with schizophrenia (SZ), and to evaluate the feasibility of this miRNA as a diagnostic marker of SZ.

**Methods:**

The serum level of miR-15a-5p in patients with SZ and healthy people was detected by RT-qPCR. ROC curve was established to evaluate the clinical diagnostic significance of miR-15a-5p in SZ. Pearson correlation coefficient was used to evaluate the correlation between miR-15a-5p level and PANSS score. Logistic regression was used to assess the risk factors of SZ. A rat model of SZ was established, and the effects of miR-15a-5p on the behavior of SZ rats were observed through water maze test and open field test.

**Results:**

The serum level of miR-15a-5p in patients with SZ was significantly increased, and ROC analysis revealed that miR-15a-5p had clinical diagnostic value in SZ. High level of miR-15a-5p was positively correlated with the positive symptom, negative symptom and general psychopathology subscore of patients. Logistic regression results showed that miR-15a-5p was a risk factor affecting the occurrence of SZ. Animal studies showed that the serum level of miR-15a-5p was elevated in the SZ rats, and inhibiting the expression of miR-15a-5p has a positive effect on improving the cognitive function and anxiety behavior of SZ rats.

**Conclusions:**

Serum miR-15a-5p is a risk factor for SZ, which is of great significance for the diagnosis of SZ.

## Background

Mental disorder refers to the disorder of brain functional activities, which leads to different degrees of mental activities such as cognition, emotion, behavior and will, and mainly includes mania, schizophrenia (SZ), anxiety, depression, and bipolar disorder (BD). There are roughly two causes of human mental disorders, one is internal, that is, genetic factors, and the other is external, that is, environmental factors [1]. SZ is a serious mental disorder of unknown etiology, and its lifetime prevalence rate is 1% [2]. Epidemiological studies showed that genetic factors are the most important factors in the pathogenesis of SZ, but the specific genetic molecular mechanism is complex and still unclear [3]. In recent years, neuroanatomical and histological features of SZ have been reported, but these findings are not uniform enough to be used as a diagnostic criterion for SZ. At present, the clinical diagnosis of SZ mainly depends on the description of symptoms, and these symptoms often overlap with other mental disorders, which may easily lead to misdiagnosis or delayed diagnosis, thus affecting the treatment and prognosis of patients with SZ [4]. Therefore, it is urgent to find biomarkers that can improve the timeliness and accuracy of clinical diagnosis.

For a long time, people thought that RNA only served as a "bridge" to extract and translate the genetic information from DNA into proteins. However, recent studies revealed that only 2–3% of the transcripts of advanced organisms encode proteins, and most of the DNA transcripts are non-coding RNA (ncRNAs) [5]. In fact, ncRNAs play a key role in regulating gene expression and maintaining physical growth and development [6]. MicroRNAs (miRNAs) are endogenous small non-coding RNA with a length of 22–25 nucleotides. They are involved in many biological processes in vivo, including cell proliferation, differentiation and apoptosis, through post-transcriptional regulation of protein-coding genes [7, 8]. Previous literature reported that miRNA is highly specific in the central nervous system [9]. The role of miRNA in the pathogenesis of mental diseases has also attracted extensive attention from scholars at home and abroad because of its abundant expression in brain tissue and its ability to maintain brain function. For example, Dinan et al. further proposed that miR-181 and miR-346 were closely related to SZ, and miR-34a and miR-144 were related to BD by combining the reported studies on the association between mental diseases and miRNA [10]. MiR-15a-5p, located in 13q14 region of human chromosome, is a member of the miR-15 family [11]. There are many members of the miR-15 family, including miR-15a, miR-15b, miR-16, miR-195, miR-424, miR-497, etc. [12]. Huang et al. established a depression-like behavior rat model by chronic unpredictable mild stress method (CUMS) and found that miR-15a-5p expression decreased in this depression model [13]. Mellios et al. found that the expression of miR-195 in SZ is enhanced [14]. Beveridge et al. reported that elevated miR-424 and miR-16 contribute to the development of SZ [15]. At present, it is not clear whether miR-15a-5p, as a member of miR-15 family, is related to SZ.

**Scheme 1:**
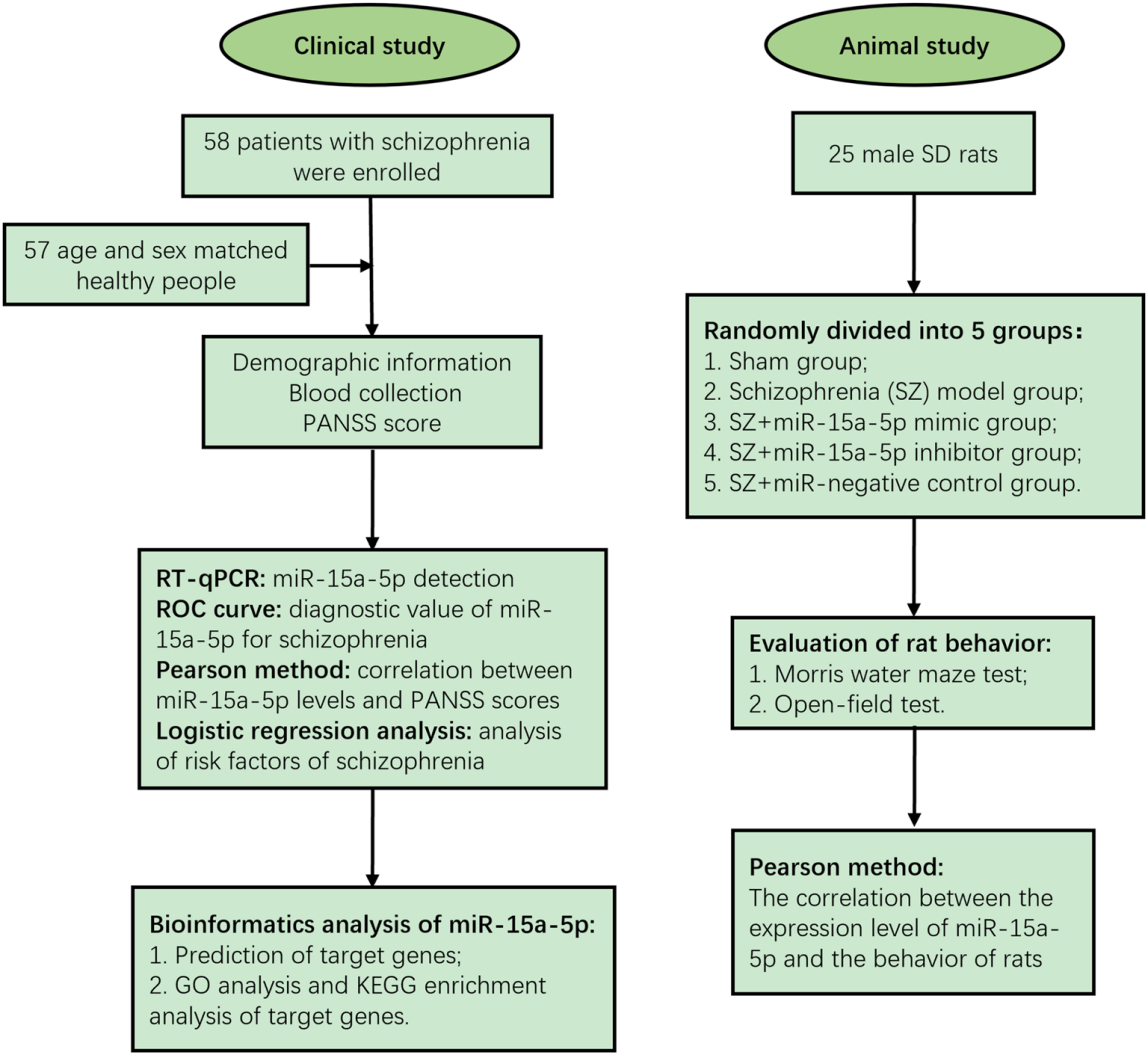
Flowchart of datasets

In this paper, the relevance between miR-15a-5p and various clinical indexes of patients with SZ was discussed by detecting the serum level of miR-15a-5p, and the clinical diagnostic significance of miR-15a-5p in SZ was evaluated. Meanwhile, the influencing factors of SZ occurrence were assessed by logistic analysis, aiming to provide some references for serum miR-15a-5p as a diagnostic biomarker of SZ. In addition, the effects of miR-15a-5p on the behavior of SZ rats were evaluated by constructing an SZ rat model (see Scheme 1), to provide experimental basis for further research on miR-15a-5p.

## Methods

### Study population and sample collection

Fifty-eight patients with SZ were selected from local hospital. Inclusion criteria: (1) the diagnosis of SZ followed the diagnostic criteria stipulated in the fifth edition of the Diagnostic and Statistical Manual of Mental Disorders IV (DSM-5) [16]; (2) complete clinical data of patients; (3) normal intelligence; (4) patients who did not take antipsychotic drugs before their first enrollment; (5) no history of mental illness or family history. Exclusion criteria: (1) patients with brain trauma; (2) patients with dysfunction of heart, liver, kidney, and other important organs; (3) accompanied by other mental disorders; (4) patients with alcohol dependence or drug abuse. Another 57 healthy subjects were selected as control group. The implementation of this study was approved by the Ethics Committee of Shenzhen Polytechnic University and conforms to the relevant provisions of the Declaration of Helsinki. All volunteers and their families were informed and signed written informed consent.

General information was collected from all volunteers, including gender, age, body mass index (BMI), family history. On the next day after inclusion, 5 mL of fasting venous blood was collected and centrifuged at 4000r/min at 4 ℃ for 10 min. The upper serum was taken and stored in a −80 ℃ refrigerator for later use.

### Determination of serum miR-15a-5p expression level

Total RNA was extracted from serum using TRIzol reagent, and cDNA was obtained by reverse transcription reaction using RNA as a template according to PrimeScript RT reagent kit instructions. RT-qPCR was carried out according to the instructions of the real-time fluorescence quantitative PCR kit. The reaction system was 20 μL, including 10 μL of SYBR Premix, 1 μL of cDNA, 8 μL of sterile purified water, and 0.5 μL of upstream and downstream primers. The cycle parameters of PCR amplification are as follows: pre-denaturation at 90 ℃ for 10 min (1 cycle), followed by 40 cycles of denaturation at 94 ℃ for 30 s, annealing at 60 ℃ for 30 s, and extension at 70 ℃ for 30 s. Each reaction was repeated for three times. U6 was used as the internal reference gene, and the relative expression level of serum miR-15a-5p was expressed by (RQ) = 2^−△△Ct^ method.

### Psychopathology data collection

The symptoms of psychopathology were assessed with the Positive and Negative Symptom Scale (PANSS) for SZ [17]. PANSS consisted of 7 positive symptoms, 7 negative symptoms and 16 general psychopathological symptoms. In this study, PANSS items P1, P3, P5, and G9 were used to estimate positive symptoms; N1, N2, N3, N4, N6, and G7 items were used to evaluate negative symptoms; P2, N5, and G11 items were used to evaluate cognitive function; P4, P7, G8, and G14 items were used to estimate arousal; and G2, G3, and G6 items were used to evaluate depressive mood.

### Animals and grouping

Twenty-five male SD rats with a body weight of (55 ± 5) g were raised in a cage alone and were given light from 7:00 to 19:00 every day to allow them to drink and eat freely. The rats began the experiment after a week of environmental adaptation. The rats were randomly divided into 5 groups, with 5 rats in each group. The blank group, also known as the sham group, received intraperitoneal injections of sodium chloride (0.9%, m/v) every day for 2 weeks. The animal models of SZ are usually constructed by intraperitoneally injection of MK-801. Rats were intraperitoneally injected with MK-801 (0.2 mg/kg) at 10:00 a.m. every day for 2 weeks to establish SZ model. In addition, the SZ model rats were divided into miR-15a-5p mimic group (SZ + 2 μM miR-15a-5p mimic), miR-15a-5p inhibitor group (SZ + 2 μM miR-15a-5p inhibitor) and miR-negative control group (SZ + 2 μM miR-NC). The above mimics, inhibitors, and negative controls of miR-15a-5p were administered by intraperitoneal injection.

### Morris water maze test

Morris water maze test was used to evaluate the cognitive function of SZ rats. The water maze is 210 cm in diameter, 51 cm in height, 10 cm in platform diameter, and 19–22 ℃ in water temperature. The water chamber was divided into four quadrants, and a platform was placed inside the chamber. The platform is placed at 1 cm below the third quadrant. The rats were first trained for 5 days, four times a day. The rats were placed at a random position in the water maze, allowing the rats to find the platform within 60 s and rest on the platform for 10 s. If the rats did not find the platform within 60 s, they were artificially placed on the platform for 10 s, and the escape latency of rats was recorded. Subsequently, the platform was removed, and the rats were placed in the first quadrant, and the times and time of passing through the original platform position in the third quadrant were recorded.

### Open field test

The open field box (100 × 100 × 50cm^3^) was divided into 9 areas. Put the camera directly above the box and connect it to the computer. Adjust the parameters to start recording. The rats were placed in the center of the box with their backs to the experimenter, and they were allowed to explore in the box for 10 min. Motor behavior in rats was quantified as total distance between spontaneous activity and rest time. In addition, the tank should be cleaned with 70% ethanol between the two tests to remove odors of other animals.

### Bioinformatics analysis

To further understand the biological function of miR-15a-5p, we tried to study the downstream target genes of miR-15a-5p by bioinformatics methods. We used TargetScan, miRDB and Martarbase databases to predict the target genes of miR-15a-5p and obtained the target genes supported by these three databases by constructing Venn diagram. Subsequently, we performed Gene ontology (GO) analysis on these target genes to determine the molecular functions (MF), cellular components (CC), and biological processes (BP) of these genes. Further, the signal pathways of potential enrichment of these target genes were briefly analyzed through Kyoto encyclopedia of genes and genomes (KEGG) enrichment analysis.

### Data analysis

SPSS 17.0 statistical software was used for statistical analysis. Quantitative data were expressed as mean ± standard deviation, and the comparison between the two groups was made by independent sample* t* test. Qualitative data were represented by *n*, and Chi-square test was chosen for comparison between the two groups. Pearson correlation coefficient analysis was used to analyze the correlation between clinical indicators and serum miR-15a-5p; Logistic regression was used to evaluate the risk factors for the occurrence of SZ; SZ was taken as the dependent variable (Assignment: SZ = 1, non-SZ = 0), and age, gender (Assignment: female = 1, male = 0), education level (Assignment: > 9 years = 1, ≤ 9 years = 0), marital status (Assignment: unmarried = 1, married = 0) and miR-15a-5p level were taken as independent variables. The diagnostic value of serum miR-15a-5p in SZ was analyzed by the receiver operator characteristic curve. *P* < 0.05 was considered a significant difference.

## Results

### Demographic characteristics

The demographic characteristics of all volunteers are shown in Table 1. There was no significant differences in sex, age, education level and marital status between the two groups (*P* > 0.05), which indicated that the two groups of cohorts were comparable.

**Table 1:** Basic clinical data of the subjects

Items	Healthy volunteers (*n* = 57)	SZ patients (*n* = 58)	*P*
Age (years)	23.58 ± 9.16	22.00 ± 8.30	0.334
Gender (Male/Female)	31/26	30/28	0.775
Education level (years)			0.517
≤ 9	28	25	
> 9	29	33	
Marital status (Married/Unmarried)	28/29	30/28	0.780
Age of onset (years)	/	20.47 ± 5.67	/

*SZ* Schizophrenia. All data are presented as mean ± standard deviation (SD) or n

### Serum expressions of miR-15a-5p and diagnostic value for SZ

**Fig. 1:**
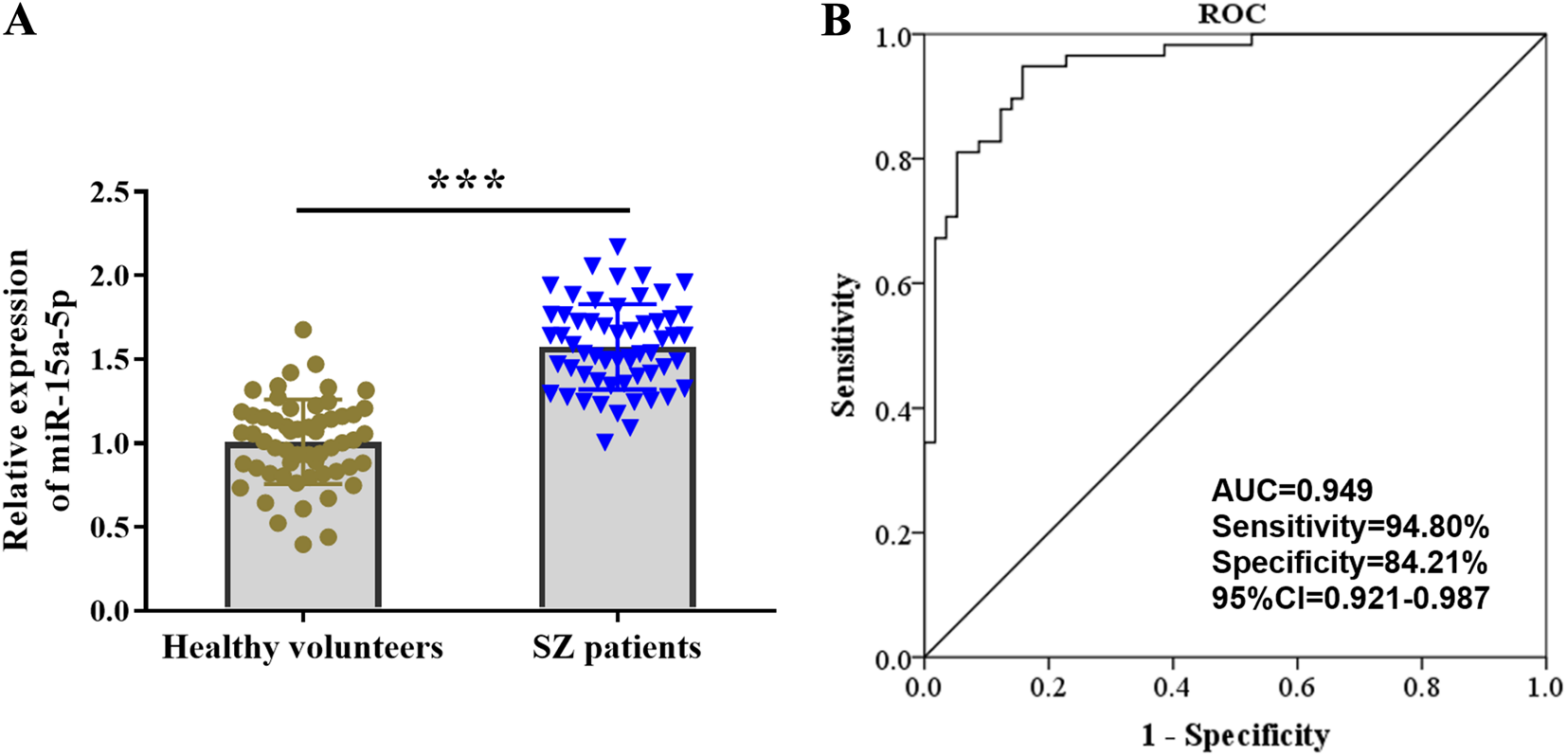
Analysis of serum level and clinical diagnostic value of miR-15a-5p. **A** RT-qPCR analysis showed that the expression of miR-15a-5p in the serum of schizophrenia patients was significantly increased compared with that of healthy controls. **B** ROC curve showed that the area under the curve of miR-15a-5p was 0.949, and the sensitivity and specificity were 94.80% and 84.21%, respectively. ^***^P < 0.001

After RT-qPCR analysis, it was found that the expression level of serum miR-15a-5p in patients with SZ was significantly higher than that in the healthy control group (Fig. 1A, *P* < 0.001), which preliminarily indicated that the abnormal expression of miR-15a-5p may be involved in the occurrence of SZ. ROC curve was used to estimate the clinical diagnostic value of serum miR-15a-5p in SZ. As shown in Fig. 1B, the area under the curve (AUC) of miR-15a-5p was 0.949, 95% CI was (0.921–0.987), and its sensitivity and specificity were 94.80% and 84.21%, respectively, which indicated that serum miR-15a-5p has a high diagnostic accuracy for SZ.

### Correlation between miR-15a-5p level and PANSS score

Besides, Table 2 exhibits the PANSS general psychopathological subscore of patients with SZ and the correlation results. Correlation results showed that in the patient group, PANSS positive subscore (*r* = 0.831, *P* < 0.001), PANSS negative subscore (*r* = 0.625, *P* < 0.001) and PANSS general psychopathology subscore (*r* = 0.809, *P* < 0.001) were positively correlated with the serum level of miR-15a-5p.

**Table 2:** Correlation between miR-15a-5p and PANSS scores

Items	Scores (n = 58)	miR-15a-5p	
		*r*	*p*
PANSS scores			
Positive symptoms	26.66 ± 6.93	0.831	< 0.001
Negative symptoms	23.84 ± 7.14	0.625	< 0.001
General psychopathology	45.31 ± 10.90	0.809	< 0.001

Data are presented as mean ± standard deviation (SD)

### Logistic regression analysis

The risk factors of SZ were evaluated by logistic regression analysis. As shown in Table 3, it could be observed that miR-15a-5p was a risk factor for the occurrence of SZ (OR = 9.462, 95% CI 4.011–22.318, *P* < 0.001).

**Table 3:** Multivariate logistic regression analysis of influencing factors in SZ patients

Factors	Multivariate analysis		
	OR	95% CI	*P*
Age (years)	1.328	0.560–3.150	0.519
Gender (*n*)	1.080	0.4555–2.564	0.861
Education degree	1.164	0.493–2.749	0.729
Marital status	1.137	0.477–2.710	0.773
MiR-15a-3p	9.462	4.011–22.318	< 0.001

*SZ* Schizophrenia, *OR* Odds Ratio, *CI* Confidence interval

### Inhibition of miR-15a-5p can improve cognitive function in SZ rats

**Fig. 2:**
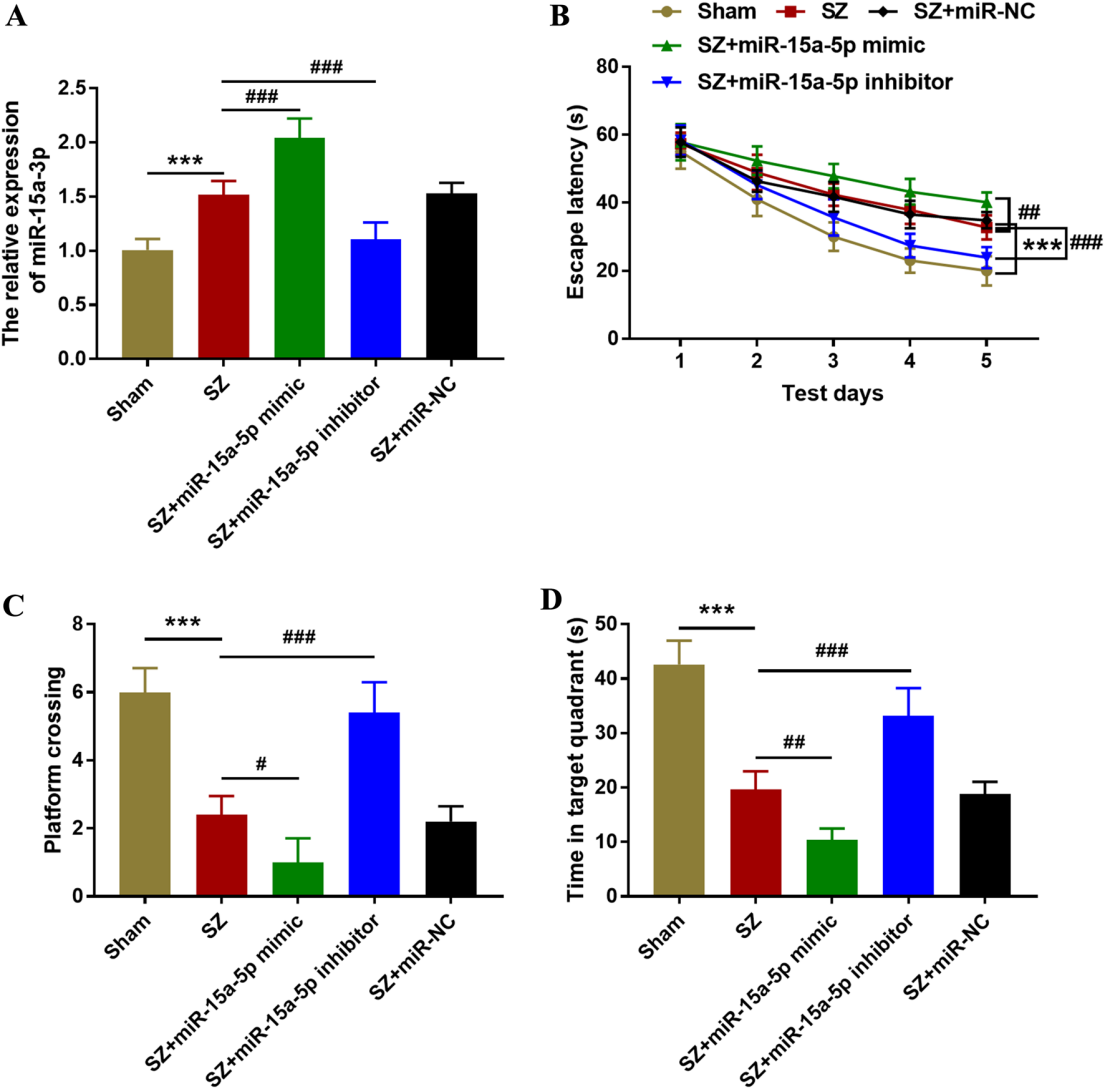
Effects of miR-15a-5p on cognitive function in rats. **A** In vivo transfection regulated the expression of miR-15a-5p. **B** The Morris water maze teat: effect of miR-15a-5p on escape latency. **C** Morris water maze teat: the influence of miR-15a-5p on the number of crossing platforms. **D** Morris water maze teat: influence of miR-15a-5p on time spent in the target quadrant. ^***^P < 0.001 vs. Sham group; ^#^*P* < 0.05, ^##^*P* < 0.01, ^###^*P* < 0.001 vs. SZ group

The regulation of miR-15a-5p in rats was achieved by in vivo transfection. The results showed that compared with the sham group, the expression of miR-15a-5p in the SZ group increased significantly. In vivo transfection of miR-15a-5p mimic or inhibitor can upregulate or downregulate the serum levels of miR-15a-5p (Fig. 2A, *P* < 0.001). The results of the water maze test showed that the escape latency of the SZ group was obviously prolonged, and the overexpression of miR-15a-5p could prolong the escape latency of the rats. On the contrary, inhibition of miR-15a-5p could significantly shorten the escape latency of the rats (Fig. 2B, *P* < 0.01). In addition, injection of MK-801 reduced the number and time of platform crossing in the target quadrant, while the inhibition of miR-15a-5p increased the number and time of platform crossing in the target quadrant (Fig. 2C, D, all *P* < 0.05).

### Inhibition of miR-15a-5p can relieve hyperactivity in rats

**Fig. 3:**
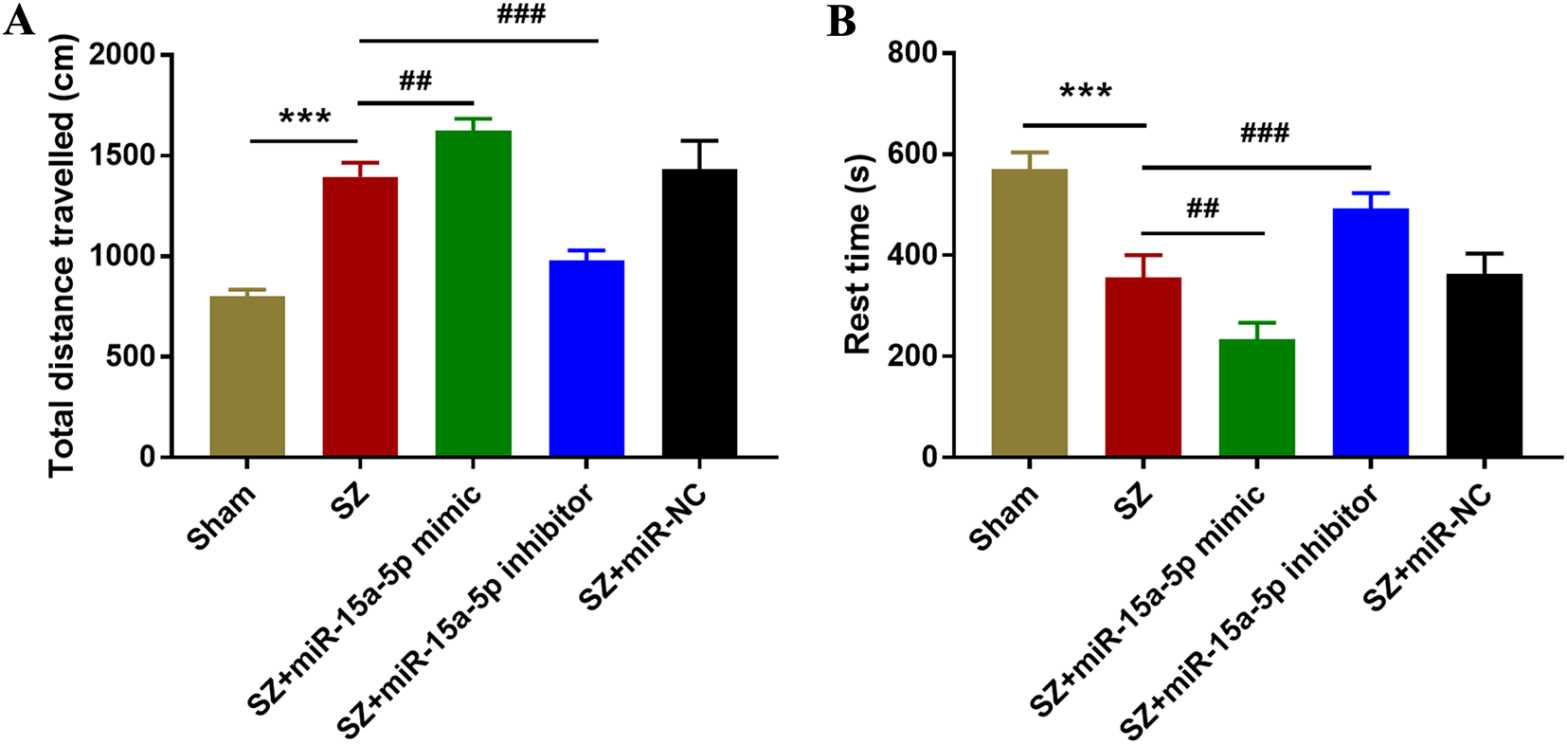
Open-field experiment. **A** Effect of miR-15a-5p on total distance travelled. **B** Effect of miR-15a-5p on rest time. ^***^P < 0.001 vs. Sham group; ^##^*P* < 0.01, ^###^*P* < 0.001 vs. SZ group

The results of open field test showed that the total distance of spontaneous activity of SZ rats increased and the rest time decreased significantly, indicating that the rats had obvious mental hyperactivity. Besides, the downregulation of miR-15a-5p could significantly reduce the total distance travelled and prolonged the rest time of SZ rat model (Fig. 3A, B, *P* < 0.01).

### Correlation analysis of serum miR-15a-5p level and behavior in rats

**Fig. 4:**
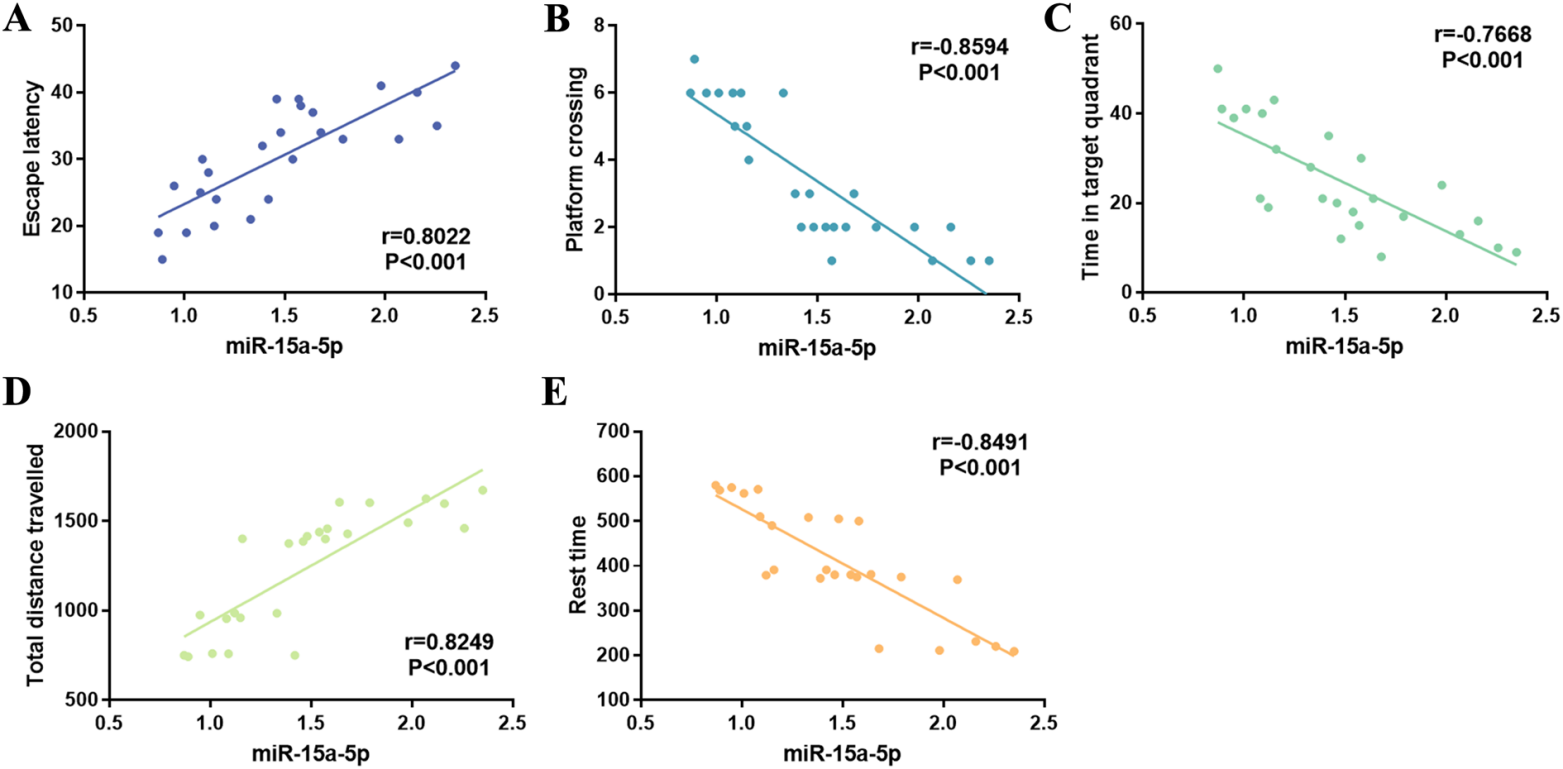
Pearson correlation analysis between the expression level of serum miR-15a-5p and the behavior of rats. **A** The level of miR-15a-5p was positively correlated with the escape latency of rats. **B** The level of miR-15a-5p was negatively correlated with the number of platform crossings. **C** The level of miR-15a-5p was negatively correlated with time in target quadrant. **D** The level of miR-15a-5p was positively correlated with total distance travelled. **E** The level of miR-15a-5p was negatively correlated with the rest time

Pearson correlation coefficient method was used to evaluate the correlation between miR-15a-p levels and behavior. The results showed that the escape latency of rats was positively correlated with the expression level of miR-15a-5p (Fig. 4A, *r* = 0.8022, *P* < 0.001). The number of crossing the platform and the residence time in the target quadrant were negatively correlated with the levels of miR-15a-5p, respectively (Fig. 4B, C, *r* = −0.8594, −0.7668,* P* < 0.001). In addition, it was observed that the total distance travelled was positively correlated with the level of miR-15a-5p (Fig. 4D, *r* = 0.8249, *P* < 0.001), while the rest time was negatively correlated with the level of miR-15a-5p (Fig. 4E, *r* = −0.8491, *P* < 0.001).

### Bioinformatics analysis of target genes of miR-15a-5p

**Fig. 5:**
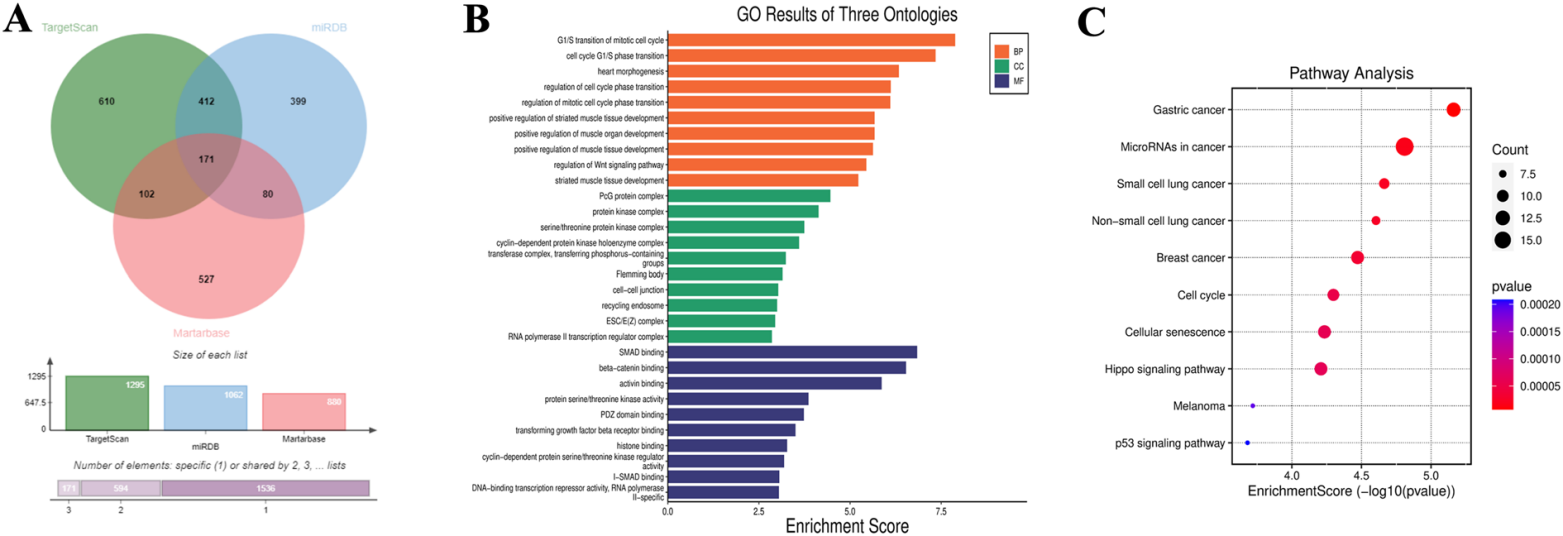
Bioinformatics analysis of miR-15a-5p target gene. **A** Venn diagram of miR-15a-5p target gene prediction. **B** GO analysis of miR-15a-5p target genes. **C** KEGG pathway analysis of miR-15a-5p target genes

The TargetScan, miRDB and Martarbase databases were selected to predict the target genes of miR-15a-5p, and the predicted results were represented by the Venn diagram (Fig. 5A). The results showed that there were 171 possible target genes of miR-15a-5p at the intersection point. The results of GO analysis are shown in Fig. 5B. Biological process (BP) analysis showed that miR-15a-5p target genes were enriched in cell cycle, mitosis, muscle organ development, striated muscle tissue development, and regulating Wnt signaling pathways. Cellular component analysis showed that these target genes were mainly concentrated in PcG protein complex, protein kinase complex, transferase complex and RNA polymerase II transcriptional regulatory complex. Molecular functional analysis indicated that the target genes were enriched in SMAD binding, transforming growth factor receptor binding, and protein serine/threonine kinase activity. KEGG analysis illustrated that these target genes were significantly gathered in p53 signaling pathway, miRNA in cancers, cell cycle, cell senescence, breast cancer, and gastric cancer (Fig. 5C).

## Discussion

SZ is a common mental illness whose etiology has not been fully elucidated. As is known to all, genetic factors play a very important role in pathogenesis. MiRNA, as a key factor regulating gene expression, has been widely concerned in the field of gene research of SZ. This study investigated the correlation between the level of miR-15a-5p in peripheral blood and SZ and verified the elevated expression of miR-15a-5p in patients with SZ by RT-qPCR. ROC analysis verified that miR-15a-5p as a diagnostic marker for SZ had high diagnostic accuracy. Correlation and regression analysis verified the correlation between miR-15a-5p and PANSS, as well as the fact that miR-15a-5p is a risk factor affecting the occurrence of SZ. In addition, animal experiments showed that the inhibition of miR-15a-5p is helpful to improve cognitive function and anxiety status of SZ rats.

Genes in the miR-15 family have been proven to play a critical role in the regulation of human cancers, such as chronic lymphocytic leukemia, liver cancer and breast cancer. Recently, many miR-15 family members have been repeatedly reported to be associated with mental illness. For example, miR-16 has been found to be differentially expressed in SZ and healthy people [18]. Li et al. reported that the expression of miR-497 increased in a female mouse model of depression, but it was not observed in a male mouse model [19]. MiR-195 expression was increased in patients with major depressive disorder [20]. In this study, RT-qPCR results showed that the level of miR-15a-5p in patients with SZ was increased. Beveridge et al. included 21 patients with SZ and 21 controls without mental illness and tested the gene expression in the gray matter of STG by chip technology. They found that the gene expression level of the miR-15 family increased, including miR-15a [15]. Another study on stress biomarkers showed significantly increased expression levels of miR-15a-5p in diseases related to psychological stress [21]. These results are consistent with those of Beveridge. Gao et al. reported that miR-15a was involved in the deletion of BDNF levels in MeCP2 regulation of developmental neurons [22]. Impaired dendritic maturation is a common pathology of many neurodevelopmental disorders, including autism and SZ [23]. BDNF is a strong supporting factor for neuron maturation [24]. Studies have shown that the reduction of BDNF protein in the brain is an important feature of MeCP2-deficient mice model [25]. Therefore, scholars speculated that the increase of miR-15a indirectly inhibits BDNF by down-regulating the expression of MeCP2, thus inhibiting neuron maturation and dendritic development, which may promote the occurrence of mental diseases. Numerous previous studies have reported miRNA as a potential biomarker in the blood of patients with mental illness. For example, Davarinejad et al. identified the abnormally expressed genes in patients with SZ through meta-analysis, and then confirmed that miR-574-5p and miR-1287 were potential biomarkers of SZ through ROC analysis [26]. In the present study, an ROC curve based on the expression level of miR-15a-5p showed that the miRNA has clinical diagnostic value in SZ. Here, we further analyzed the correlation between miR-15a-5p and PANSS score. PANSS is mainly used to assess the severity of various symptoms of SZ, and it is suitable for different types of SZ. It is a standardized rating scale widely used in clinical research [27]. This scale contains 30 items, which are divided into positive symptoms, negative symptoms, and general psychopathology categories [28]. In this study, the level of miR-15a-5p was positively correlated with positive symptom, negative symptom and general psychopathology subscore, which further proved that miR-15a-5p was related to the severity of SZ. As far as we know, early and effective diagnosis can significantly improve the therapeutic effect and prognosis of patients with SZ.

NMDA receptor antagonists such as Ketamine and MK-801 can induce a series of behavioral changes in animals, and their cognitive and sensory impairment is similar to that of human SZ. Among them, MK-801, as the most selective and non-competitive NMDA receptor antagonist, is also a classic tool for constructing SZ animal models [29]. In this study, we successfully constructed the rat model of SZ by intraperitoneal injection of MK-801. The Morris water maze results showed that the inhibition of miR-15a-5p could shorten the escape latency of SZ rats and increase the number of platform crossings and the time spent in the target quadrant, indicating that the downregulation of miR-15a-5p could improve the spatial learning and location memory ability of SZ rats. Open field test showed that inhibiting miR-15a-5p could significantly shorten the total distance travelled and increase the rest time of SZ rats, thereby improving the mental state of rats. The above experiments indicated that the reduction of miR-15a-5p had positive significance in improving the cognition and mood of SZ rats. In addition, we also explored the target genes of miR-15a-5p through three databases, and finally identified 171 possible downstream target genes including CCND1, CCND2, EZH1, AKT3, KDSR, WNT3A, CASK, CDK6, etc., among which CASK has been found to be related to central nervous system diseases and mental disorders [30]. Besides, CCND2 has been found to be associated with central nervous system diseases and mental disorders [31]. However, the expression and function of these target genes in SZ still need to be verified by further studies.

The limitations of this study are shown in two aspects. First of all, due to the difficulty in obtaining brain tissue, we selected peripheral blood samples to determine the expression level of miR-15a-5p. At present, it is not clear whether the expression level in peripheral blood is consistent with that in brain. Second, the sample size of this study is small and the source is single, which may not avoid the occurrence of selection bias. Subsequently, a multicenter large sample cohort should be selected to verify the expression trend of miR-15a-5p. Third, the exact regulatory mechanism of miR-15a-5p in SZ remains to be explored, which can be explored through animal models. Therefore, it is hoped that future studies will include more comprehensive samples for confirmation analysis, and further explore the regulation mechanism of miR-15a-5p on SZ.

## Conclusions

In conclusion, the serum level of miR-15a-5p in patients with SZ is higher than that in healthy people, suggesting that the abnormality of miR-15a-5p may be related to the occurrence of SZ. The level of miR-15a-5p is positively correlated with the severity of SZ, which has certain reference value for the diagnosis of SZ.

## Data Availability

The datasets used and/or analyzed during the current study are available from the corresponding author on reasonable request.

## References

[1] Gatt JM, Burton KL, Williams LM, Schofield PR (2015). Specific and common genes implicated across major mental disorders: a review of meta-analysis studies. J Psychiatr Res.

[2] Malavia TA, Chaparala S, Wood J, Chowdari K, Prasad KM, McClain L (2017). Generating testable hypotheses for schizophrenia and rheumatoid arthritis pathogenesis by integrating epidemiological, genomic, and protein interaction data. NPJ Schizophr.

[3] Gejman PV, Sanders AR, Duan J (2010). The role of genetics in the etiology of schizophrenia. Psychiatr Clin North Am.

[4] Chen S, Jiang H, Xu Z, Zhao J, Wang M, Lu Y (2019). Serum BICC1 levels are significantly different in various mood disorders. Neuropsychiatr Dis Treat.

[5] Trerotola M, Relli V, Simeone P, Alberti S (2015). Epigenetic inheritance and the missing heritability. Hum Genomics.

[6] Xiao L, Li Y, Zeng X, Zhou Z, Hu S, Zhang S (2020). Silencing of LOC389641 impairs cell proliferation and induces autophagy via EGFR/MET signaling in lung adenocarcinoma. Aging (Albany NY).

[7] Jiang G, Mu J, Liu X, Peng X, Zhong F, Yuan W (2020). Prognostic value of miR-21 in gliomas: comprehensive study based on meta-analysis and TCGA dataset validation. Sci Rep.

[8] Wojtukiewicz MZ, Sierko E, Hempel D, Tucker SC, Honn KV (2017). Platelets and cancer angiogenesis nexus. Cancer Metastasis Rev.

[9] Bian S, Sun T (2011). Functions of noncoding RNAs in neural development and neurological diseases. Mol Neurobiol.

[10] Dinan TG (2010). MicroRNAs as a target for novel antipsychotics: a systematic review of an emerging field. Int J Neuropsychopharmacol.

[11] Bottoni A, Piccin D, Tagliati F, Luchin A, Zatelli MC, Uberti ECD (2005). miR-15a and miR-16-1 down-regulation in pituitary adenomas. J Cell Physiol.

[12] Zhang J, Zhang D, Yan X, Jiang F (2021). The expression level and prognostic value of microRNA-15a-5p in endometrial carcinoma. Transl Cancer Res.

[13] Huang X, Yang C, Huang M (2023). Protective mechanism of the EZH2/microRNA-15a-5p/CXCL10 axis in rats with depressive-like behaviors. J Chem Neuroanat.

[14] Mellios N, Huang HS, Baker SP, Galdzicka M, Ginns E, Akbarian S (2009). Molecular determinants of dysregulated GABAergic gene expression in the prefrontal cortex of subjects with schizophrenia. Biol Psychiatry.

[15] Beveridge NJ, Gardiner E, Carroll AP, Tooney PA, Cairns MJ (2010). Schizophrenia is associated with an increase in cortical microRNA biogenesis. Mol Psychiatry.

[16] Lynall ME, Bassett DS, Kerwin R, McKenna PJ, Kitzbichler M, Muller U (2010). Functional connectivity and brain networks in schizophrenia. J Neurosci.

[17] Taraskina AE, Nasyrova RF, Zabotina AM, Sosin DN, Sosina KA, Ershov EE (2017). Potential diagnostic markers of olanzapine efficiency for acute psychosis: a focus on peripheral biogenic amines. BMC Psychiatry.

[18] Xie M, Li Z, Li X, Ai L, Jin M, Jia N (2022). Identifying crucial biomarkers in peripheral blood of schizophrenia and screening therapeutic agents by comprehensive bioinformatics analysis. J Psychiatr Res.

[19] Li B, Zhao H, Sun J (2022). Long noncoding RNA LINC00473 ameliorates depression-like behaviors in female mice by acting as a molecular sponge to regulate miR-497-5p/BDNF axis. Comput Math Methods Med.

[20] Maffioletti E, Bocchio-Chiavetto L, Perusi G, Carvalho Silva R, Sacco C, Bazzanella R (2021). Inflammation-related microRNAs are involved in stressful life events exposure and in trauma-focused psychotherapy in treatment-resistant depressed patients. Eur J Psychotraumatol.

[21] Krammer UDB, Lerch ML, Haslberger AG, Hippe B (2023). MiR-10a, miR-15a, let-7a, and let-7g expression as stress-relevant biomarkers to assess acute or chronic psychological stress and mental health in human capillary blood. Mol Biol Rep.

[22] Gao Y, Su J, Guo W, Polich ED, Magyar DP, Xing Y (2015). Inhibition of miR-15a promotes BDNF expression and rescues dendritic maturation deficits in MeCP2-deficient neurons. Stem Cells.

[23] Luo Y, Shan G, Guo W, Smrt RD, Johnson EB, Li X (2010). Fragile x mental retardation protein regulates proliferation and differentiation of adult neural stem/progenitor cells. PLoS Genet.

[24] Fargali S, Sadahiro M, Jiang C, Frick AL, Indall T, Cogliani V (2012). Role of neurotrophins in the development and function of neural circuits that regulate energy homeostasis. J Mol Neurosci.

[25] Li W, Pozzo-Miller L (2014). BDNF deregulation in Rett syndrome. Neuropharmacology.

[26] Davarinejad O, Najafi S, Zhaleh H, Golmohammadi F, Radmehr F, Alikhani M (2022). MiR-574-5P, miR-1827, and miR-4429 as potential biomarkers for schizophrenia. J Mol Neurosci.

[27] Varghese MT, Jyothi KS, Shaji KS, Rita VL (2020). Delaying clozapine: how long is too long?. Gen Psychiatr.

[28] Gopal S, Gogate J, Pungor K, Kim E, Singh A, Mathews M (2020). Improvement of negative symptoms in schizophrenia with paliperidone palmitate 1-month and 3-month long-acting injectables: results from a phase 3 non-inferiority study. Neuropsychiatr Dis Treat.

[29] Yohn SE, Conn PJ (2018). Positive allosteric modulation of M(1) and M(4) muscarinic receptors as potential therapeutic treatments for schizophrenia. Neuropharmacology.

[30] Pak C, Danko T, Mirabella VR, Wang J, Liu Y, Vangipuram M (2021). Cross-platform validation of neurotransmitter release impairments in schizophrenia patient-derived NRXN1-mutant neurons. Proc Natl Acad Sci U S A.

[31] Kerns D, Vong GS, Barley K, Dracheva S, Katsel P, Casaccia P (2010). Gene expression abnormalities and oligodendrocyte deficits in the internal capsule in schizophrenia. Schizophr Res.

